# Robotic‐assisted laparoscopic removal of dermoid cyst mimicking an enlarged cystic mass in the seminal vesicle

**DOI:** 10.1002/iju5.12477

**Published:** 2022-06-29

**Authors:** Danny Lascano, Kelly Hsieh, Bhuvi Kedia, John P Higgins, Benjamin I Chung

**Affiliations:** ^1^ Department of Surgery Westchester Medical Center Valhalla New York USA; ^2^ Department of Urology Stanford University School of Medicine Stanford California USA; ^3^ Department of Pathology Stanford University School of Medicine Stanford California USA

**Keywords:** dermoid cyst, mature, seminal vesicle histology, teratoma

## Abstract

**Introduction:**

Isolated seminal vesicle cysts not associated with Zinner syndrome is a rare disorder that can present initially with urinary obstructive symptoms or nonspecific groin pain.

**Case description:**

We present the uncommon case of a dermoid cyst mimicking a seminal vesicle cyst treated with robotic‐assisted laparoscopic seminal vesiculectomy.

**Conclusion:**

For dermoid cysts, surgical excision is the gold standard of treatment with a high cure rate and little risk of regrowth if spillage is avoided and full resection is completed. Robotic‐assisted laparoscopic surgery is a viable management option with good visualization of the anatomy.

**Abbreviations & Acronyms**
CT: computed tomography
MRI: magnetic resonance imaging

**Keynote message**

This is an unusual presentation of a dermoid cyst mimicking a seminal vesicle cyst. Understanding how this entity presents and appears and how to optimally diagnose and treat it successfully offers important lessons for the clinician. A minimally invasive approach can be successfully employed which can offer optimal visualization and control of any potential spillage.

## Introduction

Seminal vesicle cysts are a rare disorder that often presents with associated abnormalities of the kidney, vas deferens, or ectopic ureteral orifices, also known as Zinner syndrome.^1^ Isolated seminal vesicle cysts not associated with Zinner syndrome are even more umcommon and encompasses benign tumors such as fibromas, leiomyomas, cystadenomas, schwannomas, and papillary adenomas. Malignant tumors such as primary adenocarcinoma or sarcoma of the seminal vesicle are very rare. Local extension or metastasis may also present as a seminal vesicle cyst in the cases of lymphoma, prostate, bladder, or rectal cancer. Other causes of cystic findings of the seminal vesicles include abscess, hemorrhage, lymphocele, and congenital or acquired ejaculatory duct obstruction.

Teratoma is in the differential and are categorized by histological variants: mature teratoma, immature teratoma, teratoma with malignant transformation, and monodermal teratoma. Mature teratomas are sometimes present in adults but are usually seen in children. In this case, dermoid cysts are mature teratomas that are well differentiated. They present with normal serum tumor markers and are associated with lower morbidity risk. They are seen on imaging as a unilobar cyst lined with skin, forming with cutaneous adnexal structures such as sebaceous or sweat glands, hair follicles, and teeth characteristic of tissues derived from ectoderm.

## Case report

The patient is a middle‐aged African American gentleman who presented with lower urinary tract obstruction and right groin pain. He had a similar episode 8 years prior that was thought to be a right seminal vesicle abscess or cyst based on a transrectal ultrasound which was treated with aspiration and the patient's pain resolved. Pathology, cytology, and culture reports were unavailable.

**Fig. 1:**
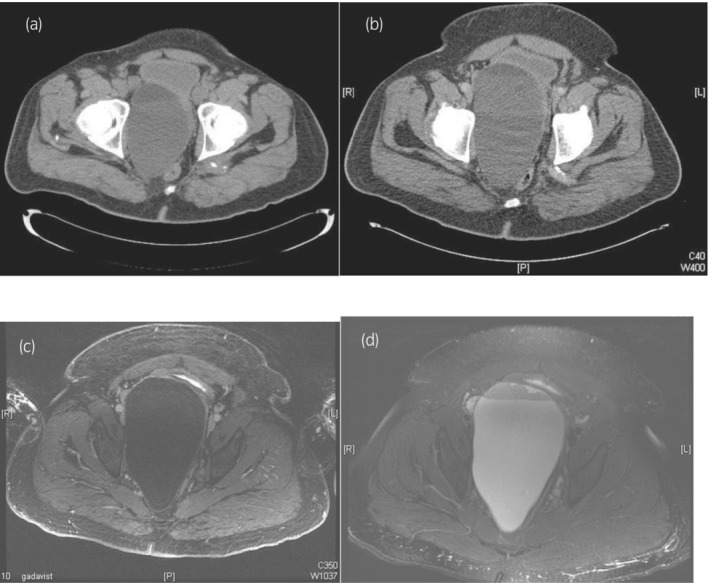
Axial images of the patient's tumor 4 years after having an aspiration of a possible abscess from his right seminal vesicle cyst. In (a) the mass measures 12.7 × 8.9 cm. In (b) 8 years after the initial presentation, the mass increases in size and measures 15.3 × 9.8 × 16.7 cm with obvious mass effect on the bladder. Finally, prior to presentation to a urologist, a MRI scan with gadolinium was done which shows a 17.7 × 12.0 × 11.1 cm mass that in (c) that is non‐enhancing in comparison the bladder which enhances anteriorly in the T1 phase and fat filled in the T2 phase as shown in (d).

He was lost to follow‐up until he presented with recurrence of his right groin pain. A CT scan demonstrated a large retroperitoneal mass measuring 12.7 × 8.9 cm felt to be a seminal vesicle cyst (Fig. 1a). He was then lost to follow‐up and presented 4 years later for recurrent discomfort. CT imaging showed an increase in size of the mass to 15.4 × 9.9 × 16.8 cm (Fig. 1b). Follow‐up MRI imaging showed a non‐enhancing mass (Fig. 1c) and fat‐containing cystic lesion (Fig. 1d) thought to be a right seminal vesicle cyst which had grown to 17.7 × 12.0 × 11.1 cm.

In the clinic, the patient reported incomplete bladder emptying, moderate frequency, mild intermittency, severe urgency, severely weak flow of urination, and nocturia two times per night despite taking 0.4 mg of tamsulosin daily. He had an American Urological Association symptom score of 20 and quality of life score of 5.

On physical examination, a digital rectal examination was unremarkable, although the examination was limited due to body habitus as the patient had a body mass index of 35.1. His creatinine and laboratory values were within normal limits.

Given the patient's obstructive urinary symptoms and discomfort, the patient opted for robotic‐assisted laparoscopic seminal vesiculectomy. A pure laparoscopic approach could also have been chosen as well. Several single institution studies support the decreased morbidity, decreased length of stay, and better exposure attained by minimally invasive techniques such as laparoscopic surgery for seminal vesiculectomy although no evidence other than case studies support similar benefits for robotic approaches.^2^

### Surgery

The patient was placed in dorsal lithotomy. Cystoscopy at the start of the case did not reveal any masses or diverticula. Bilateral ureteral catheters were placed for ureteral identification during the case. Ports were placed in a configuration identical to robot assisted laparoscopic prostatectomy with the da Vinci Xi system (Intuitive Surgical, Sunnyvale, CA, USA). The instruments used were also identical for that of robotic prostatectomy, including two left hand fenestrated graspers and a right hand monopolar scissors.

**Fig. 2:**
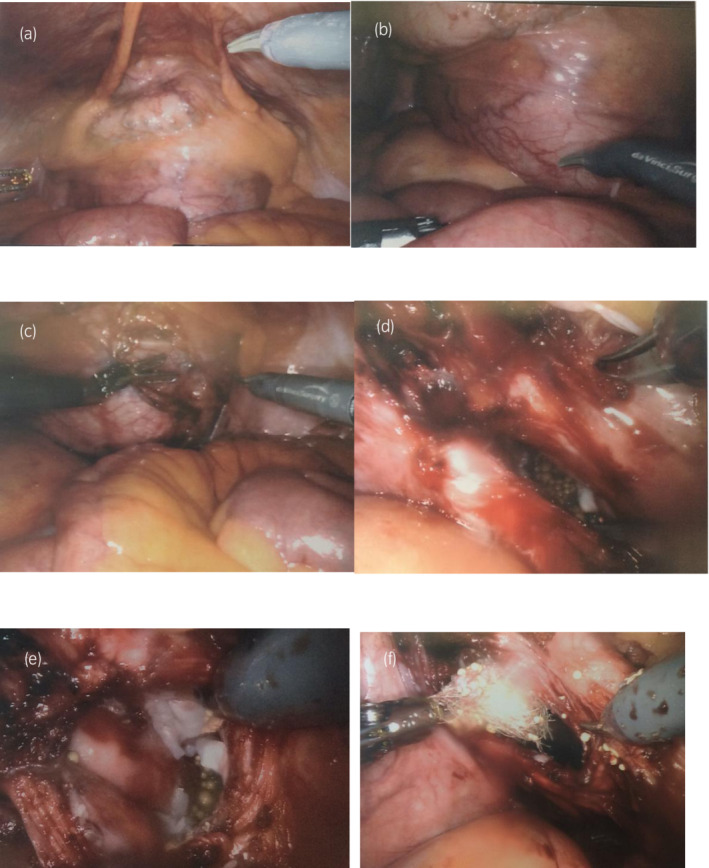
Intraoperative findings showing a large mass abutting the bladder (a) before fluid filling. The bladder was then filled with fluid (b) and the cyst was punctured (c) to facilitate the surgery. The mass revealed skin glands (d), cartilage (e), and hair (f).

The delineation between the large cyst and bladder was difficult after the bladder was drained (Fig. 2a) so it was instilled with sterile saline (Fig. 2b). The peritoneal reflection overlying the cyst posteriorly was then dissected free. To avoid unintentional uncontrolled spillage during dissection, approximately 500 mL of turbid fluid was aspirated from the cyst without spillage. The cyst was dissected and showed irregular lining with yellow tinted material in addition to skin glands, cartilage, and hair (Fig. 2c–f). Dissection of the right ureter from the cyst was completed after decompression of the cyst. The surgical bed was irrigated with saline. Sigmoidoscopy was performed due to the difficulty of separating the structures from the rectum and did not reveal any evidence of injury. The left ureteral catheter was removed and the right ureteral catheter was exchanged to a 6‐French right JJ stent given the extensive ureterolysis on that side. The specimen was entrapped in a bag and removed, the peritoneum was irrigated with saline, and incisions were closed in standard fashion.

**Fig. 3:**
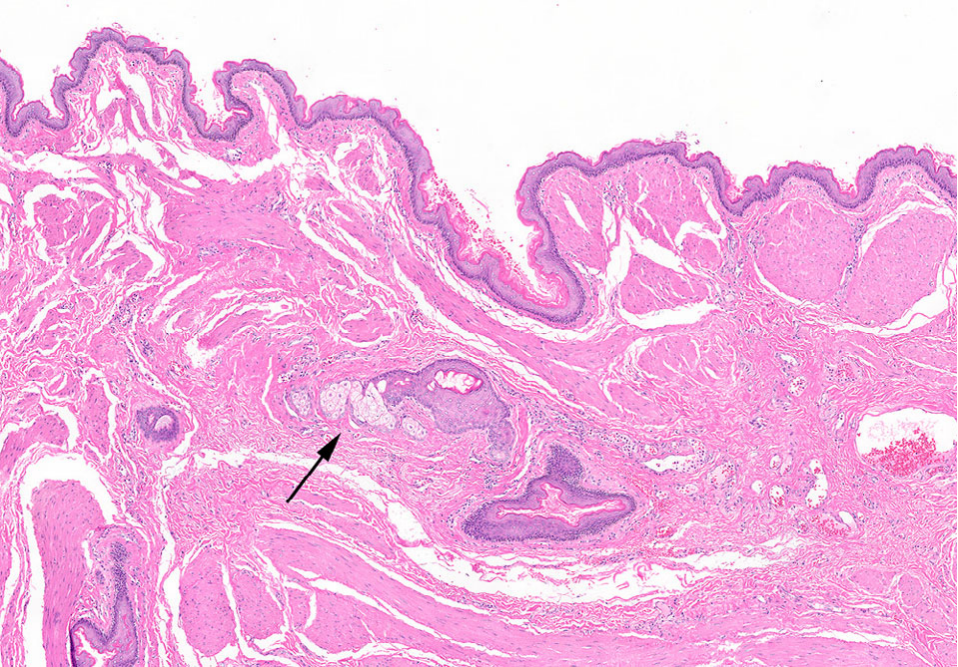
Histologic cross section of dermoid cyst wall with arrow pointing to sebaceous gland within the keratinizing squamous epithelium, pathognomonic of a dermoid cyst.

Patient's postoperative course was unremarkable. Cytology revealed bland anucleate keratinocytes and rare intact, mature squamous cells consistent with a squamous lined cyst. Cultures grew out *Bacteroides fragilis* and other anaerobic gram‐positive cocci including *Peptostreptococcus*, *Finegoldia*, *Peptoniphilus*, and *Anaerococcus*. Pathology revealed that the seminal vesicle was normal and distinct from the cystic structure. The cystic structure measured 9.5 × 8.2 × 3.1 cm and was lined by a keratinizing squamous epithelium with scattered sebaceous units consistent with a dermoid cyst or mature cystic teratoma. (Fig. 3) The arrow points to a sebaceous gland located within the keratinizing squamous epithelium, which is a finding pathognomonic for dermoid cyst.

As the patient was not compliant with routine follow‐up, we do not know if the dermoid cyst excision resulted in an improvement in the patient's urinary symptoms.

## Discussion

This case represents the third reported case of a dermoid cyst localized in the pelvis of a male patient and the first reported case of a dermoid cyst mimicking a seminal vesicle cyst reported in the literature.^3^, ^4^ In these previous cases, the dermoid cyst similarly underwent curative surgical excision, although like in our case, the diagnosis was not able to be made preoperatively. The findings were very similar to ours, with the cyst filled with a dense, yellowish, cheesy material. This material is derived from the keratinizing epithelium admixed with the various additional elements present, such as sebaceous glands and even hair. Unlike our case, these previous cases utilized an open surgical approach, whereas ours utilized a minimally invasive robotic approach. Our case does lend evidence that a minimally invasive approach can safely and successfully be applied in this situation.

Close follow‐up is warranted when spillage occurs during resection of the mass since malignant transformation of a reimplanted dermoid cyst can occur in the retroperitoneum. Moreover, leakage of the sebaceous material can result in chemical granulomatous peritonitis; this can be avoided by placing the mass in an impermeable bag prior to removal. Complete resection is usually curative.

## Author contributions

Danny Lascano: Data curation; writing – original draft. Kelly Hsieh: Writing – review and editing. Bhuvi Kedia: Writing – review and editing. John P Higgins: Formal analysis; supervision; writing – review and editing.

## Conflict of interest

Benjamin I. Chung has received honoraria from Intuitive Surgical.

## Approval of the research protocol by an Institutional Reviewer Board

N/A.

## Informed consent

N/A.

## Registry and the Registration No. of the study/trial

N/A.
